# Innate immunity and antibiotic activity in resistant bacterial infections in immunocompromised hosts

**DOI:** 10.3389/fcimb.2026.2002377

**Published:** 2026-10-29

**Authors:** Yao Sun, Yiming Guo, Haizhen Cui, Yueyao Jiang, Qian Yu

**Affiliations:** 1 Department of Pharmacy, China-Japan Union Hospital of Jilin University, Changchun, China; 2 School of Pharmaceutical Sciences, Jilin University, Changchun, China

**Keywords:** adjunctive therapy, antimicrobial resistance, bacterial persistence, host defense, host–antibiotic interface, immunocompromised hosts, innate immunity, pharmacokinetics/pharmacodynamics

## Abstract

Antimicrobial resistance (AMR) is particularly difficult to manage in immunocompromised hosts, in whom impaired bacterial containment and clearance can coexist with restricted treatment options, altered drug exposure, and anatomically complex infection sites. Susceptibility testing and pharmacokinetic/pharmacodynamic (PK/PD) assessment guide antibiotic treatment, while host defenses help determine whether injured bacteria are cleared. This Review examines the host–antibiotic interface in resistant bacterial infection, with emphasis on how innate immunity contributes to antibacterial activity in immunocompromised populations. We distinguish quantitative and qualitative defects in neutrophils, monocyte hyporesponsiveness, complement impairment, barrier disruption, and combined host deficits, and consider how bacterial burden, infection compartment, and protected intracellular or extracellular populations modify drug–host interactions. Experimental studies show that immune function influences bacterial growth, clearance, and observed antibiotic exposure–response relationships. Antibiotic-induced changes in bacterial morphology, microbial-product release, and immune recognition may promote clearance or inflammatory injury depending on pathogen, burden, timing, and host state. Adjunctive approaches, including colony-stimulating factors, interferon-γ, innate agonists, pathogen-specific antibodies, antibody–antibiotic conjugates, and phage–antibiotic combinations, vary substantially in mechanism and strength of supporting evidence. Clinical assessment of adjunctive strategies requires evidence of improved infection outcomes as well as biological activity. We propose a research-oriented framework integrating antibacterial exposure, bacterial phenotype and burden, infection compartment, and host immune function to guide mechanistic investigation, while retaining effective antibiotic treatment and source control when indicated as the foundations of clinical care.

## 1 Introduction

Antimicrobial resistance (AMR) complicates bacterial infection by limiting effective treatment options, but patient immune function also affects outcomes. In patients with hematologic malignancies, transplantation, or defined immune defects, impaired bacterial containment can coexist with prior antibiotic exposure, colonization by resistant organisms, tissue injury, and altered drug disposition. Clinical cohorts of carbapenem-resistant Enterobacterales (CRE) bloodstream infection during neutropenia and carbapenem-resistant *Klebsiella pneumoniae* (CRKP) infection after liver transplantation illustrate why resistant infection is especially concerning in these patients (Satlin et al., 2016; Dang et al., 2026).

Antibiotic selection and adequate exposure remain central to treatment. Phenotypic susceptibility testing, commonly based on the minimum inhibitory concentration (MIC), evaluates bacterial susceptibility under standardized *in vitro* conditions (Clinical and Laboratory Standards Institute, 2024). Pharmacokinetic/pharmacodynamic (PK/PD) analysis relates antimicrobial exposure to antibacterial effect (Saporta et al., 2026). Standard susceptibility testing does not reproduce active complement, phagocytes, tissue architecture, or effector recruitment to the infected site. Treatment response also depends on drug delivery to the infection site, bacterial burden, and host defenses.

Antibiotic exposure, bacterial characteristics, and host defenses influence one another. Neutrophils, monocytes/macrophages, complement, epithelial barriers, and host-defense molecules influence bacterial containment and elimination. Antibiotics, in turn, alter bacterial viability, morphology, physiology, and the release or exposure of material recognized by these defenses (Kolaczkowska and Kubes, 2013; Kaufmann and Dorhoi, 2016; Wolf et al., 2017; Mondeme et al., 2023). Impaired host defenses may leave viable bacteria after antibiotic treatment, whereas antibiotic-triggered immune activation may contribute both to microbial control and to inflammatory tissue injury.

Host–antibiotic interactions occur in both susceptible and resistant infections. AMR can amplify the consequences of impaired clearance and can alter bacterial susceptibility to host effectors through changes in surface structure, physiology, or stress responses.

Patients with immunocompromise differ in the host defenses that are impaired. Chemotherapy-associated neutropenia, inherited phagocyte dysfunction, pharmacological complement blockade, and post-transplant immunosuppression involve different combinations of effector abundance, cellular competence, recruitment to infected tissue, and barrier integrity. Studies of sepsis-associated dysfunction, diabetes, and ageing provide complementary examples of impaired antibacterial functions. Experimental comparisons across different immune states demonstrate why host function can alter bacterial trajectories and the apparent contribution of antibiotic-mediated killing (Drusano et al., 2010; Thorsted et al., 2023; Saporta et al., 2026).

This Review focuses on innate immune mechanisms that influence antibiotic activity and bacterial clearance in immunocompromised hosts. Adaptive immune responses are discussed where relevant to these interactions, including antibody-mediated opsonization and neutralization of therapeutic phages. Vaccine development and microbiome-directed therapies are outside its scope.

Our recent Review evaluated the clinical evidence for newer antibiotics in immunocompromised patients, including pivotal trial representation, treatment outcomes, and applicability to high-risk populations (Sun et al., 2026). The present Review examines how innate immune defects interact with antibiotic-induced bacterial changes to influence clearance. We focus on phagocytes, complement, bacterial burden, and protected infection compartments, drawing on experimental, pharmacodynamic, and selected clinical studies. We also evaluate adjunctive strategies and identify testable questions for their clinical development. **Figure 1** summarizes the interactions considered in this Review.

**Figure 1:**
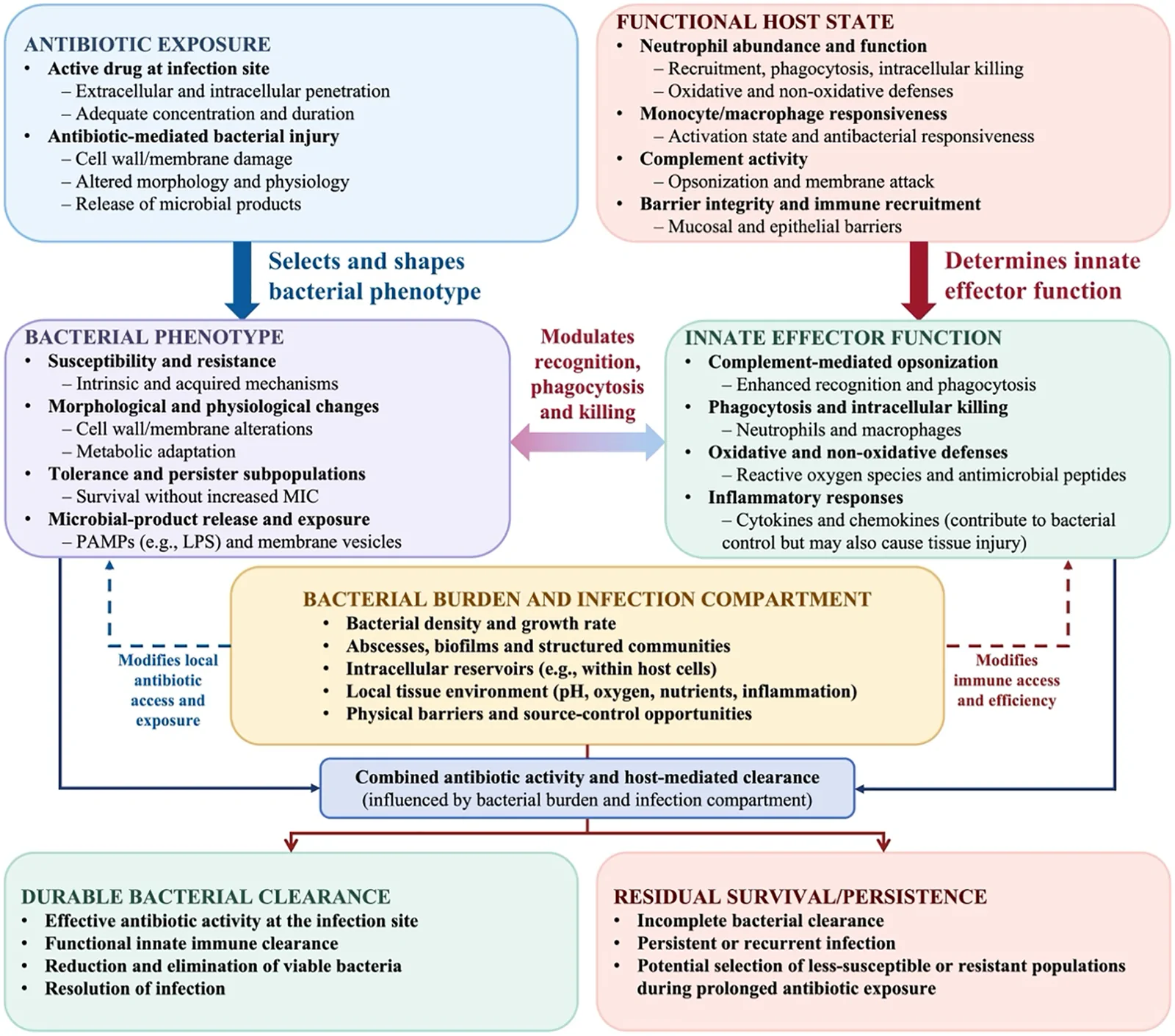
Host and antibiotic determinants of bacterial clearance.

Antibiotic exposure causes bacterial injury and alters bacterial morphology, physiology, and susceptibility to host defenses. Innate immune effectors contribute to bacterial clearance through complement-mediated opsonization, phagocytosis, intracellular killing, and oxidative and non-oxidative mechanisms. Their activity depends on the functional host state, including neutrophil availability, monocyte/macrophage responsiveness, complement activity, and tissue recruitment. Bacterial burden and infection compartment modify antibiotic exposure and immune access, particularly in abscesses, biofilms, and intracellular reservoirs. These interacting factors influence whether treatment achieves sustained bacterial clearance or leaves surviving bacterial populations. Residual survival under continued antibiotic selection may facilitate the emergence or expansion of resistant variants. Solid arrows represent biological interactions, whereas dashed arrows indicate modifying effects of bacterial burden and infection compartment.

## 2 Scope and evidence approach

This is a structured narrative Review. We searched PubMed/MEDLINE primarily for studies of antibacterial exposure, innate immune effectors, bacterial infection and protected compartments, and adjunctive or host-directed interventions. We prioritized primary mechanistic and clinical studies and used relevant reviews for background information and to identify additional references. We performed additional focused searches to identify newer clinical and translational studies.

We considered three features of each study: the experimental setting, whether it directly examined resistant bacteria, and the immune status of the host. Findings from laboratory experiments, animal studies, quantitative models, and human studies differ in how directly they apply to patient care. Independently, a study may directly examine a resistant organism or address resistance-independent biology, and may involve a defined immune deficit or a broader population. We therefore distinguish evidence directly involving resistant pathogens and defined immune defects from studies providing broader clinical or mechanistic context.

The Review is organized around mechanisms at the host–antibiotic interface rather than around individual bacterial groups. Gram-positive and Gram-negative pathogens are discussed as mechanistic examples where they inform interactions among antibacterial exposure, bacterial phenotype, host state, and infection compartment; mycobacterial evidence is included selectively when directly relevant to these mechanisms. Organism-specific resistance, tolerance, or persistence mechanisms are discussed when they change how bacteria respond to antibiotics or host defenses.

Relevant adaptive mechanisms include antibody-mediated engagement of Fc receptors and complement, T-cell support of macrophage function, and antibody-mediated neutralization of therapeutic phages (Chaplin, 2010; Kaufmann and Dorhoi, 2016; Dedrick et al., 2021). Colonization and prior antimicrobial exposure are included where they inform the clinical context of resistant infection. Selected preventive studies are included when they inform the mechanisms or evidence supporting particular adjunctive strategies. Resistance, tolerance, persistence, and biofilm biology are considered when they contribute to residual bacterial survival during treatment. We did not aim to identify every eligible study or formally assess study quality or the certainty of evidence.

## 3 Immunocompromised hosts and functional immune deficits

Immunocompromised patients differ in the type and severity of their antibacterial immune defects. Understanding these differences requires considering the underlying disease, immunosuppressive treatment, timing relative to chemotherapy or transplantation, immune recovery, epithelial injury, and infection anatomy. Routine blood counts can identify numerical deficits, but a normal count does not establish preserved recruitment, opsonization, phagocytosis, or intracellular killing. Functional measurements should therefore be interpreted within the clinical context in which infection occurs (Chaplin, 2010; Kolaczkowska and Kubes, 2013).

Among adult neutropenic patients with hematologic malignancies, Satlin et al. identified 43 CRE bloodstream infections; prior CRE culture, selected antibiotic exposures, and glucocorticoid use were associated with CRE bacteremia, and active treatment was frequently delayed (Satlin et al., 2016). In 48 liver transplant recipients with CRKP infection, intra-abdominal infection and septic shock were associated with 90-day mortality, whereas the estimate for effective antibiotic treatment within 48 hours was imprecise and not statistically significant (Dang et al., 2026).

**Table 1** maps functional phenotypes to representative clinical contexts and candidate measurements, and **Figure 2** summarizes the principal innate domains considered below.

**Table 1:** Functional host-defense phenotypes in immunocompromised and adjacent clinical contexts.

Phenotype	Clinical contexts	Limiting function	Potential consequence for bacterial clearance	Candidate measurements
Neutrophil deficiency	Cytotoxic chemotherapy; marrow failure; hematologic malignancy; congenital neutropenia	Insufficient effector abundance	Reduced host contribution to bacterial clearance; experimental exposure–response relationships may differ across immune states	ANC trajectory; depth and duration of neutropenia; marrow recovery
Qualitative phagocyte dysfunction	Inherited phagocyte defects; diabetes, ageing, and selected inflammatory states as adjacent functional contexts (Simell et al., 2011; Sapey et al., 2017; Roy et al., 2022)	Recruitment, phagocytic uptake, or intracellular killing	Reduced clearance of viable or antibiotic-injured bacteria despite preserved circulating cell counts	Chemotaxis, phagocytosis, oxidative burst, or direct bacterial-killing assays
Sepsis-associated monocyte hyporesponsiveness	Selected patients with persistent hyporesponsiveness during or after sepsis	Monocyte activation and cytokine responsiveness	May reduce the host contribution to bacterial control; quantitative effects on antibiotic-mediated bacterial clearance in humans remain undefined	Serial standardized mHLA-DR (Demaret et al., 2013); ex vivo stimulation assays
Complement impairment	Inherited complement defects; pharmacological C5 blockade (Socie et al., 2019)	Pathway-dependent opsonization or membrane attack	May reduce complement-dependent antibacterial activity in selected pathogen–drug combinations	CH50/AH50; pathway-specific complement activity; relevant antimicrobial exposure where appropriate
Barrier disruption and impaired tissue access	Mucositis; wounds and burns; indwelling devices; devitalized tissue; postoperative or inadequately controlled foci	Anatomical containment and effective immune or antimicrobial access	Protected bacterial burden may persist when antibiotics or immune cells have restricted access (Hofstee et al., 2020; Pettygrove et al., 2021)	Source imaging; device or wound assessment; local microbiology; assessment of source-control opportunities
Combined deficits	Hematologic malignancy; transplantation; complex treatment-related immunosuppression (Satlin et al., 2016; Dang et al., 2026)	Multiple host-defense functions, often accompanied by variable antibacterial exposure	Heterogeneous and dynamically changing effects on bacterial containment and clearance	Medication history, immune recovery, infection source, bacterial burden, and antibacterial exposure assessed together

ANC, absolute neutrophil count; mHLA-DR, monocyte human leukocyte antigen-DR; CH50/AH50, classical/alternative pathway functional complement assays; PK/PD, pharmacokinetics/pharmacodynamics.

**Figure 2:**
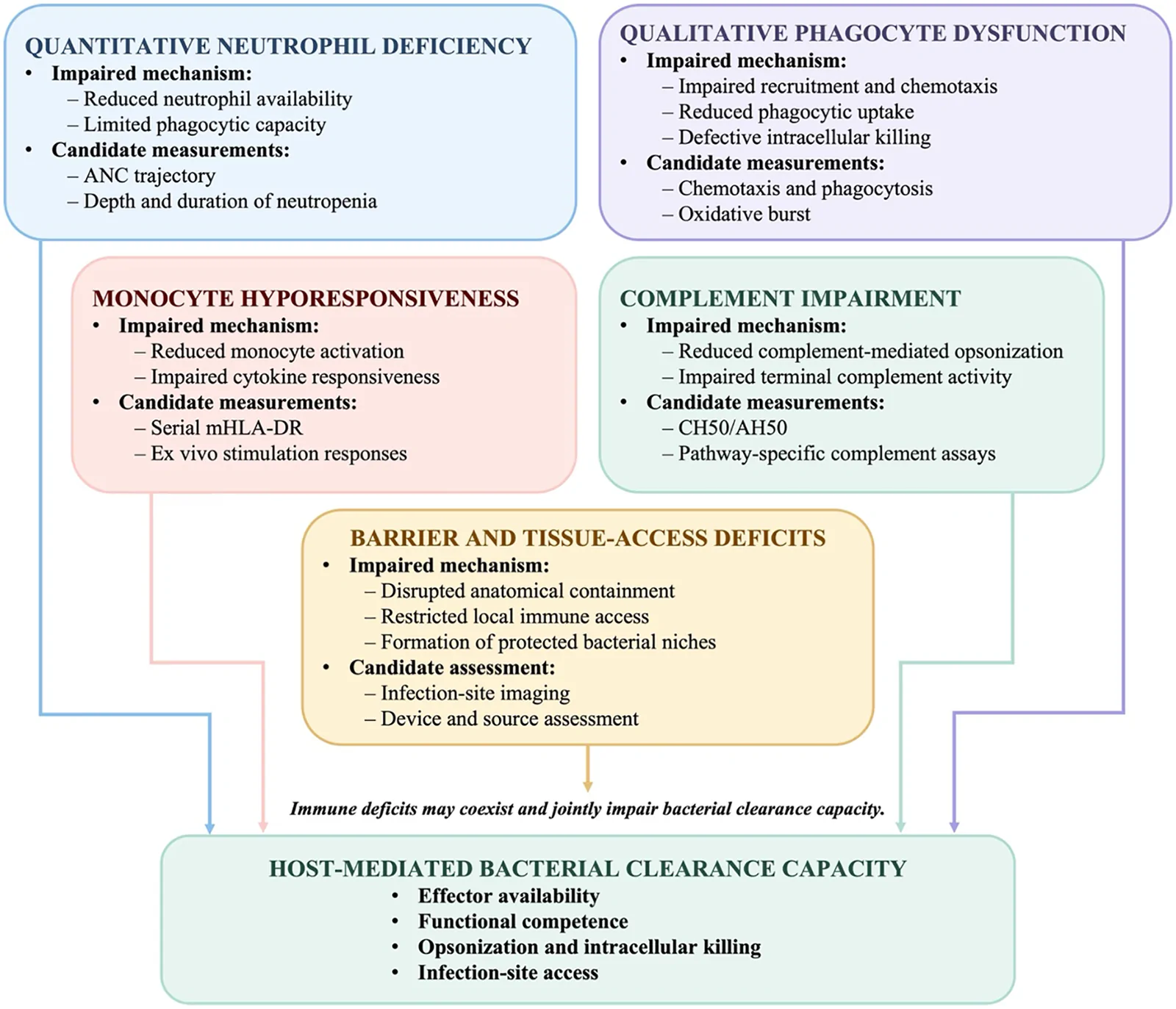
Functional innate immune deficits, antibacterial mechanisms, and candidate measurements.

Distinct innate immune deficits affect bacterial clearance through different mechanisms. Quantitative neutrophil deficiency reduces available phagocytic capacity, whereas qualitative phagocyte dysfunction impairs recruitment, bacterial uptake, and intracellular killing. Monocyte hyporesponsiveness reduces cellular activation and cytokine responses, while complement impairment affects opsonization and terminal complement activity. Barrier disruption and protected infection compartments further restrict immune access to bacterial populations. Candidate measurements include absolute neutrophil count (ANC) trajectories, functional phagocyte assays, monocyte human leukocyte antigen-DR (mHLA-DR), ex vivo stimulation responses, complement assays (CH50/AH50), and infection-site assessment. These deficits may coexist and jointly impair host-mediated bacterial clearance.

### 3.1 Quantitative neutrophil deficiency

Profound and prolonged neutropenia reduces the number of effectors available for antibacterial defense. Circulating counts describe abundance, while tissue recruitment and per-cell activity also contribute to clearance (Kolaczkowska and Kubes, 2013). The relationship between neutrophil capacity and bacterial burden is discussed in Section 4.1.

Clinically accessible measures include the absolute neutrophil count (ANC), depth and duration of neutropenia, and trajectory of count recovery. These variables describe neutrophil numbers rather than how well individual cells function and should be distinguished from functional measurements of chemotaxis, phagocytosis, oxidative burst, or direct bacterial killing.

### 3.2 Qualitative phagocyte dysfunction

Neutrophil abundance does not necessarily reflect cellular function. Defects in recruitment, phagocytosis, oxidative killing, or related effector processes can occur despite preserved circulating counts. Studies in diabetes and ageing illustrate selected defects in recruitment, phagocytosis, and other antibacterial functions (Simell et al., 2011; Sapey et al., 2017; Roy et al., 2022).

Diabetic wounds provide an example of impaired recruitment. In a murine infected-wound model, defective formyl-peptide-receptor signaling delayed neutrophil trafficking, whereas activation of an alternative chemotactic pathway with chemokine C-C motif ligand 3 (CCL3) improved recruitment, infection control, and healing (Roy et al., 2022). Related work showed reduced generation of bioactive pathogen-associated molecular patterns (PAMPs) and weaker early Toll-like receptor (TLR) signaling despite greater bacterial burden (Roy et al., 2024), illustrating how impaired early antibacterial responses can coexist with subsequent persistent inflammation. The direction and timing of immune modulation are therefore likely to matter.

Ageing provides a second example in which preserved counts can coexist with impaired opsonophagocytic function. Older adults showed reduced functional anti-pneumococcal antibody activity and neutrophil-mediated killing despite preserved complement activity (Simell et al., 2011). Neutrophil migratory accuracy also declined with age and infection severity; high-dose simvastatin improved migratory accuracy in healthy older adults in a randomized crossover study and ex vivo during milder respiratory infection, but not in pneumonia associated with sepsis (Sapey et al., 2017).

Qualitative phagocyte dysfunction may affect recruitment, opsonization, uptake, intracellular killing, or sustained effector activity. Testing these functions separately can reveal the step limiting bacterial clearance and guide the choice of functional assays.

### 3.3 Sepsis-associated monocyte hyporesponsiveness

Inflammatory and immunosuppressive responses can change over the course of sepsis. In some patients, sustained innate hyporesponsiveness is characterized by reduced monocyte human leukocyte antigen-DR (mHLA-DR) expression and impaired ex vivo cytokine production (Poehlmann et al., 2009; Drewry et al., 2016). Drewry et al. found lower mHLA-DR expression in nonsurvivors, while Cajander et al. observed delayed recovery in *S. aureus* bloodstream infection and persistently low expression among patients with secondary bacteremia or death (Drewry et al., 2016; Cajander et al., 2018).

Clinical interpretation also depends on reliable measurements. Multicenter application of mHLA-DR requires standardized preanalytics, calibration, and reporting as antibodies bound per cell because numerical thresholds cannot be assumed to transfer unchanged across analytical platforms (Demaret et al., 2013).

### 3.4 Complement impairment

Complement impairment provides a clear example of pathogen-selective innate immune deficiency. Pharmacological C5 blockade with eculizumab markedly increases susceptibility to meningococcal infection; in long-term pharmacovigilance, meningococcal infection remained the principal mechanism-related infectious risk despite reported vaccination in most affected patients (Socie et al., 2019). This provides direct human evidence that loss of a defined innate pathway can generate pathogen-specific vulnerability.

Complement can also modify antibacterial activity. Membrane attack complex (MAC)-mediated outer-membrane permeabilization increases access of otherwise excluded antimicrobial proteins to Gram-negative bacteria (Heesterbeek et al., 2021), while engineered complement recruitment restored azithromycin susceptibility in two resistant *Neisseria gonorrhoeae* isolates with efflux-pump overexpression (Bettoni et al., 2021). Complement-dependent effects on antibiotic activity vary with the pathogen and infection compartment.

### 3.5 Barrier disruption and combined deficits

Damage to epithelial or tissue barriers can allow bacteria to enter, increase bacterial burden, and help protected bacterial communities form before immune cells can clear them. Relevant settings include mucositis, wounds and burns, devitalized tissue, indwelling devices, and other forms of epithelial injury.

Abscess and device models show that delayed neutrophil access can allow organized *Staphylococcus aureus* communities to resist later clearance (Hofstee et al., 2020; Pettygrove et al., 2021). Section 6 examines these protected populations in detail.

Functional deficits also coexist and evolve during treatment. Hematologic malignancy may combine neutropenia, mucosal injury, and treatment-related immune dysfunction, whereas transplantation may combine pharmacological immunosuppression, operative tissue injury, devices, and a persistent anatomical focus. An older patient with sepsis may instead display qualitative phagocyte abnormalities, monocyte hyporesponsiveness, and altered antibacterial exposure. These patients should not be classified solely as “immunocompromised” or “not immunocompromised.” Longitudinal assessment should distinguish factors that facilitate bacterial entry or colonization from those that limit clearance of an established infection.

## 4 Host effects on antibacterial pharmacodynamics

### 4.1 Bacterial burden and available effector capacity

Mechanism-based antibacterial PK/PD models commonly represent bacterial growth and drug-mediated killing, sometimes with additional bacterial subpopulations, whereas host immune activity may be omitted, simplified, or represented through aggregate model terms (Thorsted et al., 2023; Saporta et al., 2026). Neutropenic animal infection models are widely used for antibacterial PK/PD characterization because suppression of host immunity reduces confounding between drug-mediated and immune-mediated bacterial killing and permits more consistent bacterial growth (Saporta et al., 2026). Suppressing immunity isolates drug-mediated killing under experimental conditions, whereas patients often have changing immune function and antibiotic exposure.

Early murine studies showed that granulocyte-mediated bacterial clearance depends on both effector abundance and bacterial burden (Drusano et al., 2010, 2011b; Guo et al., 2011). Guo et al. modeled a nonlinear relationship between circulating neutrophil count and bacterial killing using *Acinetobacter baumannii* pneumonia data and validated the model with *Pseudomonas aeruginosa* (Guo et al., 2011). In thigh and lung infection models, granulocyte-mediated killing became saturated as bacterial burden increased, indicating that clearance capacity depends on the relationship between bacterial burden and available granulocyte activity rather than simply on a binary “neutropenic versus non-neutropenic” state (Drusano et al., 2010, 2011b). Granulocytes also modified antibiotic-mediated killing across drug-pathogen systems: they markedly enhanced tedizolid activity against *Staphylococcus aureus* in a thigh model (Drusano et al., 2011a), while in *P. aeruginosa* pneumonia, plazomicin-mediated burden reduction enabled additional granulocyte-mediated killing once bacterial density fell below the range associated with granulocyte saturation (Drusano et al., 2014). Together, these findings suggest that granulocyte-mediated killing can become saturated as bacterial burden increases, reducing its relative contribution to clearance.

Thorsted and colleagues separated neutrophil-mediated killing into phagocytosis and intracellular digestion in a model integrating multiple *in vitro* and *in vivo* datasets (Thorsted et al., 2023). Both processes depended on neutrophil availability and became less efficient as the number of bacteria within each neutrophil increased. The model incorporated neutrophil influx, reinforcing the distinction between circulating abundance and tissue-level activity. Together, these studies support the hypothesis that antibiotic-mediated burden reduction can allow available granulocytes to contribute more effectively to clearance; the magnitude and timing remain dependent on the model, pathogen, and site.

Reducing bacterial burden through source control may also improve the contribution of available host effectors. Section 6 discusses abscesses, biofilms, and intracellular reservoirs as distinct barriers to bacterial clearance.

### 4.2 Immune state and experimental exposure response

The measured effect of an antibiotic partly depends on how bacterial numbers change without treatment. When host immunity contributes substantially to bacterial control, untreated animals may exhibit slower net growth or even bacterial clearance, compressing the measurable separation among antibiotic exposures. In neutropenic animals, weaker immune clearance can make differences between antibiotic doses more apparent. The treatment-versus-control difference therefore depends on the untreated host-mediated bacterial trajectory (Saporta et al., 2026).

Saporta and colleagues compared meropenem activity in neutropenic, intermediately immunosuppressed, and immunocompetent mice with *Klebsiella pneumoniae* lung infection (Saporta et al., 2026). Immune state changed bacterial trajectories and model-derived exposure-response relationships. The fraction of the dosing interval during which free concentrations exceeded the MIC (fT>MIC) showed the strongest correlation with predicted bacterial counts among the tested PK/PD indices. At higher exposures, the model predicted a larger drug-attributable reduction relative to the corresponding untreated trajectory under neutropenic than immunocompetent conditions, while host-mediated clearance contributed more strongly to total bacterial control in immunocompetent animals.

The study used a meropenem-susceptible collection strain, *K. pneumoniae* DSM116099 (MIC 0.032 mg/L), rather than a resistant clinical isolate. It examined one murine lung model, and the final model represented immune state categorically after continuous granulocyte scaling gave a poorer fit (Saporta et al., 2026). The findings illustrate how immune state changes measured antibiotic pharmacodynamics in this experimental system.

Comparisons of tedizolid in neutropenic ICR and immunocompetent CBA/J mice with *Streptococcus pneumoniae* lung infection yielded different exposure–response relationships. Exposures associated with stasis and 1- and 2-log killing were higher in the neutropenic model, although the difference was smaller than that previously reported in a *Staphylococcus aureus* thigh model. The investigators discussed differences in infection model and the substantial pulmonary penetration of tedizolid as possible explanations (Abdelraouf and Nicolau, 2017).

Experimental PK/PD models often isolate a defined immune defect, such as neutrophil depletion, whereas clinical immunocompromise commonly involves overlapping abnormalities in neutrophil abundance and function, epithelial barriers, and treatment-related immune suppression that evolve over time. Differences in species, bacterial strains, infection sites, drug disposition, and treatment duration further limit quantitative extrapolation. Consequently, changes in exposure–response relationships or PK/PD targets observed across animal immune states cannot be translated directly into human dose adjustments, which could lead to inadequate antibacterial exposure or unnecessary toxicity. These findings instead provide hypotheses for human studies incorporating antibiotic exposure, immune trajectories, and microbiological outcomes.

Antibiotic exposure at the infection site cannot necessarily be inferred from plasma concentrations or total concentrations measured in tissue homogenates. Tissues comprise distinct extracellular, intracellular, and subcellular compartments in which antimicrobial distribution may differ substantially; consequently, homogenization can obscure the pharmacologically relevant concentration available to bacteria (Mouton et al., 2008). Measurement of unbound drug in defined compartments, including interstitial fluid by microdialysis, can therefore provide a more direct characterization of local antimicrobial exposure (Brunner et al., 2005; Mouton et al., 2008). Drug exposure at the infection site and local tissue conditions should therefore be assessed separately from plasma PK.

Increasing systemic exposure may add toxicity without improving delivery to a protected compartment or restoring an absent immune function. Investigation of persistent infection should therefore assess local drug access and host function alongside exposure.

### 4.3 Relationship to conventional PK/PD and host-directed therapy

Conventional antibacterial PK/PD remains the basis for relating drug exposure to susceptibility and antibacterial effect. This review also considers how bacterial burden, immune function, and infection site affect that relationship.

Host-directed therapy (HDT) aims to strengthen host defenses, disrupt host processes that help pathogens survive, or reduce tissue injury. It is one possible response to a demonstrable limiting host function. Host-directed adjuncts should be evaluated in relation to a defined biological target and tested for additional benefit alongside appropriate antibacterial treatment.

## 5 Reciprocal interactions between antibiotics, bacteria, and innate defenses

### 5.1 Bacterial injury and immune recognition

Antibiotics can kill bacteria, change their shape, and alter the bacterial components that host immune cells encounter. β-Lactam-induced morphology depends in part on penicillin-binding-protein (PBP) selectivity and drug concentration. PBP3-directed β-lactam activity can produce elongated Gram-negative filaments, whereas the predominantly PBP2-active carbapenem imipenem produces rounded forms and is associated with a different pattern of endotoxin release (Jackson and Kropp, 1992; Yokochi et al., 2000; Buijs et al., 2008). These morphological responses are immunologically relevant: in an experimental *Pseudomonas aeruginosa* infection, imipenem-induced rounded cells were more readily phagocytosed than ceftazidime-induced filamentous cells, and the morphological differences were accompanied by differences in circulating endotoxin (Yokochi et al., 2000).

The amount and inflammatory activity of material released during bacterial killing depend on the antibiotic class and concentration, the organism, and the surrounding biological conditions. Experiments with log-phase *Escherichia coli* showed antibiotic- and concentration-dependent endotoxin release in RPMI 1640 and tumor necrosis factor (TNF) and interleukin-6 responses in whole blood. At 50 times the MIC, ceftazidime and ciprofloxacin produced higher responses than imipenem and gentamicin (Prins et al., 1995).

Human comparisons of ceftazidime and imipenem produced less consistent results. In severe melioidosis, ceftazidime treatment was associated with significantly greater systemic endotoxin after the first antibiotic dose than imipenem, despite similar pretreatment endotoxin and cytokine measures (Simpson et al., 2000). By contrast, randomized studies in Gram-negative urosepsis and nosocomial pneumonia did not consistently show treatment-related differences in endotoxin or cytokine responses (Luchi et al., 2000; Maskin et al., 2002), illustrating the influence of pathogen, infection site, sampling time, and clinical context.

Gram-positive bacteria display analogous behavior. β-Lactam exposure can markedly increase the release of biologically active lipoteichoic acid (LTA) and peptidoglycan from *Staphylococcus aureus* (van Langevelde et al., 1998; Lotz et al., 2006). Supernatants from flucloxacillin-exposed *S. aureus* delayed neutrophil apoptosis, increased interleukin-8 release, and enhanced phagocytic activity, effects that were markedly reduced after depletion of LTA (Lotz et al., 2006).

Resistance mechanisms themselves can also reshape immune recognition. In methicillin-resistant *Staphylococcus aureus* (MRSA), mecA induction reduces peptidoglycan cross-linking, increasing peptidoglycan degradation and inflammasome-mediated interleukin-1β (IL-1β) responses and exacerbating immunopathology, whereas subinhibitory β-lactam exposure can upregulate a SarA-controlled lipoprotein-like gene cluster that promotes Toll-like receptor 2 (TLR2)-dependent inflammatory responses and pathogenicity (Muller et al., 2015; Shang et al., 2019).

Antibiotic exposure can also change bacterial membrane-vesicle release: in carbapenem-resistant *Acinetobacter baumannii*, sub-MIC ceftazidime induced more bacterial membrane vesicles with greater lipopolysaccharide (LPS) content and inflammatory and virulence activity than imipenem (Chiu et al., 2020).

In a *Pseudomonas aeruginosa* wound model, systemic tobramycin reduced bacterial burden and increased bioactive LPS, TLR1/2/4 expression, tumor necrosis factor-α (TNF-α) and interleukin-1β, inflammatory-cell infiltration, and neutrophil activation in immunocompetent C57BL/6 mice. Severely immunodeficient NOD-scid IL2Rγ-null (NSG) mice showed weaker bacterial control and no comparable secondary inflammatory response. Topical LPS or N-formyl-methionyl-leucyl-phenylalanine (fMLP) improved infection control in NSG mice, and neutrophil-depletion experiments established a role for neutrophils in antibiotic- and LPS-associated protection (Padmakumari et al., 2026). Tobramycin was administered one hour before wounding and inoculation, and LPS or fMLP was applied before infection, so the study examined early infection control. NSG mice have combined innate and adaptive defects; the depletion experiments specifically implicate neutrophil function (Padmakumari et al., 2026).

These findings suggest a reinforcing sequence: antibiotics kill some bacteria and release bacterial material, innate immune cells respond, and those cells help clear additional bacteria. The same PAMP-driven response can cause inflammatory injury, making the balance between bacterial clearance and tissue toxicity a central question for future intervention studies.

### 5.2 Host effects on bacterial access and killing

Neutrophils influence antibiotic activity in several ways. Opsonophagocytosis removes viable or antibiotic-injured organisms, while oxidative burst and granule-associated antimicrobial mechanisms contribute to bacterial killing (Kolaczkowska and Kubes, 2013). Neutrophil extracellular traps (NETs) can immobilize bacteria and may contribute to extracellular antimicrobial activity, although their direct bactericidal effect is context-dependent; mature *Staphylococcus aureus* biofilms can induce leukocidin-dependent NET formation while remaining resistant to NET-associated clearance (Brinkmann et al., 2004; Bhattacharya et al., 2018).

A striking pharmacological example is the ability of nafcillin, despite the conventional β-lactam resistance of MRSA, to increase bacterial susceptibility to killing by selected host-defense peptides, neutrophils, whole blood, and keratinocytes (Sakoulas et al., 2014). The interaction is not invariably synergistic. In an established *S. aureus* abscess model, neutrophil depletion enhanced cefazolin-mediated bacterial killing, while brief exposure of *S. aureus* to neutrophils reduced PBP2 expression, suggesting that neutrophil exposure can modify bacterial antibiotic targets and thereby alter β-lactam activity within the infection compartment (Bamberger et al., 2002).

Complement contributes to antibacterial defense through opsonization, inflammatory signaling, and MAC activity. In Gram-negative bacteria, MAC-mediated outer-membrane permeabilization can allow otherwise excluded antimicrobial proteins, including lysozyme and human group IIA secreted phospholipase A2, to reach their targets and can enhance bacterial degradation and killing within neutrophils (Heesterbeek et al., 2021).

This permeability effect can also modify antibiotic susceptibility. Engineered C4BP-IgM, which promotes classical-pathway complement activation on *Neisseria gonorrhoeae*, restored azithromycin susceptibility in two resistant clinical isolates with MtrC-MtrD-MtrE efflux-pump overexpression; the proposed mechanism was MAC-mediated membrane perturbation that increased antibiotic access to intracellular targets (Bettoni et al., 2021). Earlier work similarly showed that polymyxin B treatment rendered several serum-resistant Gram-negative bacilli susceptible to complement-dependent killing, although the effect varied among species (Fierer and Finley, 1979).

These findings show that complement can help kill bacteria and, by disrupting bacterial membranes, increase the activity of selected antibiotics. The same mechanism predicts weaker complement-dependent activity during terminal-complement blockade or at infection sites with limited complement activity.

Macrophages and monocytes contribute to antibacterial defense through phagocytosis, intracellular killing, cytokine signaling, and coordination of local inflammatory responses (Kaufmann and Dorhoi, 2016). Host cells can also shelter viable bacteria during antibiotic exposure, as shown for intracellular *Staphylococcus aureus* persisters in infected-cell experiments (Peyrusson et al., 2020). Section 6 examines the resulting delivery and clearance barriers.

Host antimicrobial molecules attack bacteria in ways that differ from conventional antibiotics. LL-37 can inhibit *Pseudomonas aeruginosa* biofilm development at concentrations below those required to inhibit planktonic growth (Overhage et al., 2008), whereas calprotectin restricts manganese and zinc availability and thereby compromises *S. aureus* defenses against oxidative stress (Kehl-Fie et al., 2011). These examples illustrate that innate antibacterial activity can operate through biofilm- and nutrient-related mechanisms that complement antibiotic-mediated killing.

**Figure 3** summarizes the potential contributions of antibiotic-associated innate responses to clearance and tissue injury.

**Figure 3:**
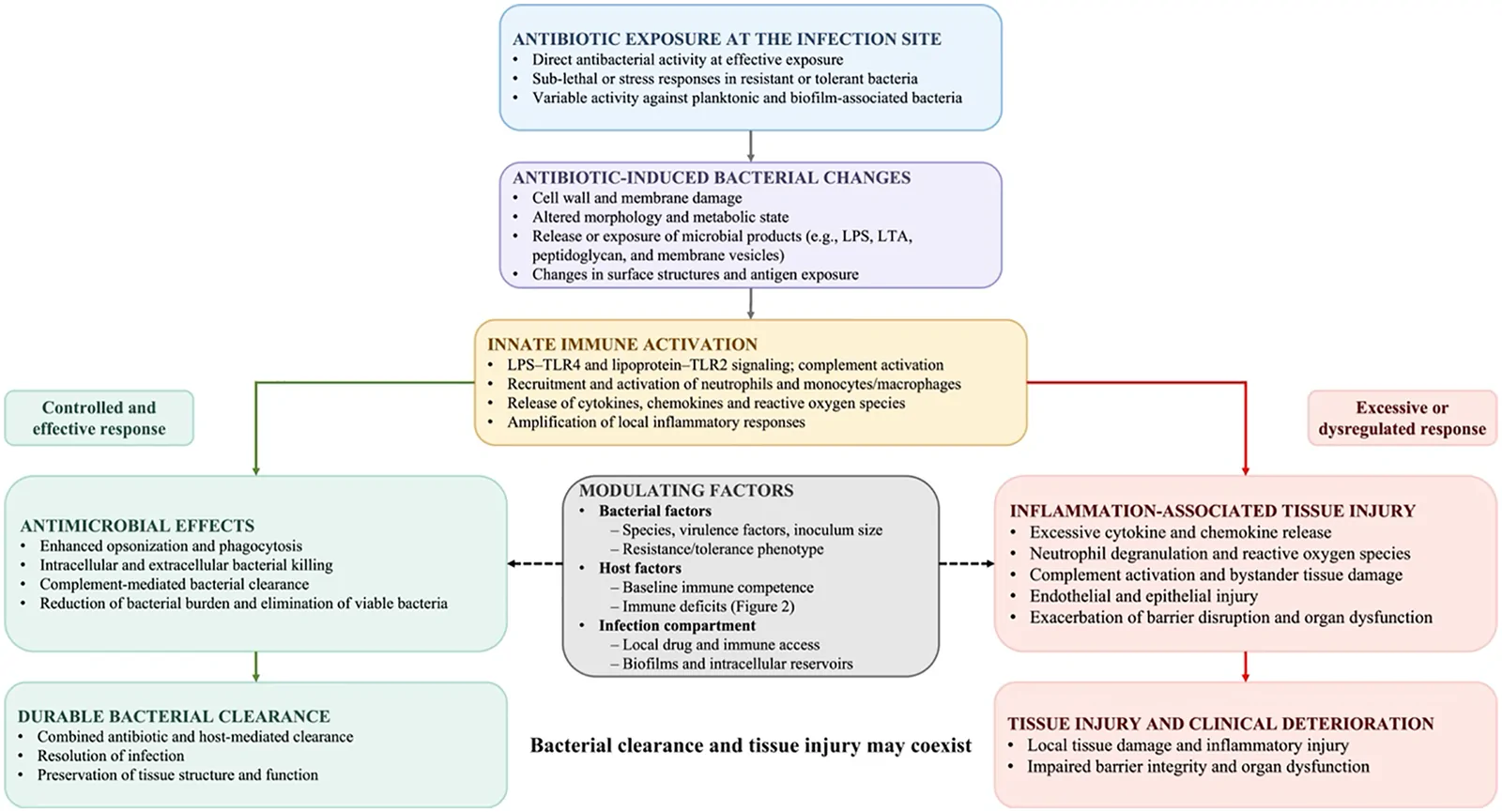
Bacterial clearance and tissue injury after antibiotic-associated immune activation.

Antibiotic exposure causes bacterial injury and alters bacterial morphology, physiology, and the release or exposure of microbial products. These changes can influence pattern-recognition receptor signaling, complement activation, and the recruitment and activity of neutrophils and monocytes/macrophages. Antibacterial responses promote opsonization, phagocytosis, and bacterial killing, while excessive inflammatory activation can contribute to oxidative damage, barrier disruption, and tissue injury. The relative contributions of these processes are influenced by bacterial characteristics, host immune function, bacterial burden, and infection compartment. Bacterial clearance and inflammatory tissue injury may occur simultaneously.

### 5.3 Bacterial clearance and inflammatory injury

Antibiotic-associated innate responses can contribute to both bacterial clearance and tissue injury. Evaluating their net effect requires measuring viable bacterial burden and host injury together.

Restoring a missing immune function is different from increasing inflammation where immune activity is already high. The appropriate intervention may involve restoration of an absent function, improved tissue access, or control of an anatomical focus. The timing of intervention, bacterial burden, and local tolerance for inflammation shape its potential benefit and risk.

Host-acting antibacterial compounds provide a related approach by modifying host-cell processes involved in intracellular survival or antibacterial activity (Liu et al., 2022; Thom and D'Elia, 2024; Zheng et al., 2026). Their relevance depends on a retained, accessible host pathway and reproducible benefit alongside antibiotics. This is particularly important when the pathway needed by an adjunct is itself impaired by the patient’s disease or treatment.

Resistance, tolerance, and persistence differ in their growth and killing responses to antibiotics (Balaban et al., 2019). **Supplementary Figure S1** summarizes these phenotypes and their possible interactions with host clearance.

**Table 2** summarizes the principal mechanisms, evidence settings, and uncertainties in translating them to human infection compartments.

**Table 2:** Mechanisms and uncertainties at the host–antibiotic interface.

Component	Mechanism and representative evidence	Possible consequence	Evidence setting	Human compartment uncertainty
Burden and neutrophils	Capacity-limited phagocytosis and digestion (Drusano et al., 2010; Drusano et al., 2011b; Guo et al., 2011; Thorsted et al., 2023)	Burden reduction may increase the contribution of available effectors	Animal and quantitative models	Combine circulating counts with relevant measures of tissue recruitment and effector function.
Morphology and microbial products	Antibiotic-dependent morphology, LPS, LTA, peptidoglycan and vesicle release (Jackson and Kropp, 1992; Prins et al., 1995; van Langevelde et al., 1998; Buijs et al., 2008; Chiu et al., 2020)	Changed recognition may promote clearance or inflammation	*In vitro/ex vivo*; animal; small human studies	Local density, product clearance and inflammatory responsiveness vary; systemic readouts may miss the site response (Luchi et al., 2000; Simpson et al., 2000; Byl et al., 2001; Maskin et al., 2002).
Complement permeability	MAC injury increases access to antimicrobial proteins; engineered recruitment can sensitize selected resistant gonococci (Fierer and Finley, 1979; Bettoni et al., 2021; Heesterbeek et al., 2021)	Enhanced activity in specific pathogen–drug systems	*In vitro/ex vivo*	Assess complement activity in the compartment relevant to the infection.
Intracellular compartments	Cell-associated *S. aureus* can persist; antibody–antibiotic conjugates can deliver intracellular payloads (Lehar et al., 2015; Peck et al., 2019; Peyrusson et al., 2020)	Active intracellular exposure may be needed in addition to extracellular activity	Cell and animal studies; phase 1 human development	Antibiotic access depends on cell type, subcellular localization, and payload release.
Host-defense molecules	LL-37 can inhibit biofilm development; calprotectin restricts metals and weakens oxidative-stress defense (Overhage et al., 2008; Kehl-Fie et al., 2011)	May complement antibiotic effects on bacterial physiology	*In vitro* and animal	Local concentrations, pH, ionic conditions, binding and nutrient availability modify activity.
Spatial architecture	Biofilms, abscesses and delayed recruitment protect bacterial populations (Bhattacharya et al., 2018; Hofstee et al., 2020; Pettygrove et al., 2021)	Source control may reduce protected burden and improve access	Structured *in vitro* and animal models	Variation in human lesion maturity, perfusion, matrix, and effector access requires infection-site assessment.
Inflammatory response	Antibiotic injury changes immune-active material and signaling (Moore et al., 2003; Lotz et al., 2006; Muller et al., 2015; Shang et al., 2019; Padmakumari et al., 2026)	Clearance and tissue injury can increase together	Cellular and animal studies	Local tolerance and cytokine responses may differ from systemic measures; therapeutic windows remain undefined.
Host immune state and AMR evolution	Impaired immune clearance permits expansion of less-susceptible bacterial variants and subsequent resistance selection during ciprofloxacin-treated *A. baumannii* pneumonia (Huo et al., 2022).	Treatment failure before conventional resistance thresholds are reached, followed by selection of more resistant populations	Experimental mouse pneumonia with serial bacterial evolution	Whether similar evolutionary trajectories occur in immunocompromised patients remains to be established

LPS, lipopolysaccharide; LTA, lipoteichoic acid; MAC, membrane attack complex.

## 6 Protected bacterial populations and persistent infection

Impaired host clearance can prolong bacterial survival during antibiotic treatment. Residual survival may reflect inadequate local drug exposure, high bacterial burden relative to available effector capacity, protected abscess or biofilm architecture, or intracellular persistence and tolerance (Mouton et al., 2008; Drusano et al., 2010, 2011b; Hofstee et al., 2020; Peyrusson et al., 2020; Pettygrove et al., 2021). Extended survival increases opportunities for recurrence and, during continued antimicrobial exposure, for selection within the surviving population.

Host immune function can also influence the evolution of antibiotic resistance by determining which bacterial variants survive during treatment. Huo et al. examined this relationship through serial passage of *Acinetobacter baumannii* in a ciprofloxacin-treated mouse pneumonia model. In cyclophosphamide-treated, neutrophil-depleted mice, bacterial variants carrying mutations affecting the efflux regulator AdeL expanded during antibiotic exposure, whereas immunocompetent mice restricted their expansion. Notably, antibiotic treatment failure occurred while most bacteria remained below the clinical ciprofloxacin resistance breakpoint. Continued passage subsequently selected populations with higher levels of resistance (Huo et al., 2022). These findings demonstrate how impaired host clearance can promote both bacterial persistence during treatment and the subsequent selection of resistant bacterial lineages. Cyclophosphamide also affects immune populations other than neutrophils.

Understanding bacteria that survive inside cells requires examining bacterial uptake, drug delivery within the cell, and bacterial physiology. *Staphylococcus aureus* can enter reversible nondividing states during intracellular antibiotic exposure (Peyrusson et al., 2020). An antibody-antibiotic conjugate that releases a rifamycin-class payload after intracellular proteolysis improved bacterial elimination in preclinical models (Lehar et al., 2015). Human development is discussed in Section 7.3.

Extracellular biofilms and abscesses can restrict antibiotic and phagocyte access through spatial organization. In a three-dimensional *in vitro* model, *Staphylococcus aureus* abscess communities surrounded by a fibrin pseudocapsule displayed marked antibiotic tolerance and resisted neutrophil clearance; plasmin pretreatment before neutrophil exposure reduced bacterial counts within the communities (Hofstee et al., 2020). These findings support investigation of lesion architecture and local drug access. When an infected device or removable focus sustains bacterial burden, source control can improve access to the affected compartment. Intracellular reservoirs require a different delivery strategy, with active drug reaching bacteria within host cells.

**Supplementary Figure S2** separates intracellular reservoirs from extracellular biofilm and abscess communities.

Studies of intracellular persistence should specify the host-cell type, subcellular location, and active drug exposure in the relevant compartment. Studies of extracellular protection should describe lesion or matrix architecture, local drug access, and access by immune cells.

Colonization, ongoing infection, microbiological persistence, and recurrent acquisition should be distinguished in studies of residual bacterial survival.

## 7 Adjunctive strategies and the functions they require

Adjunctive strategies differ in their mechanisms, required host functions, and available evidence. **Figure 4** summarizes the mechanisms and **Table 3** compares intervention-specific evidence.

**Figure 4:**
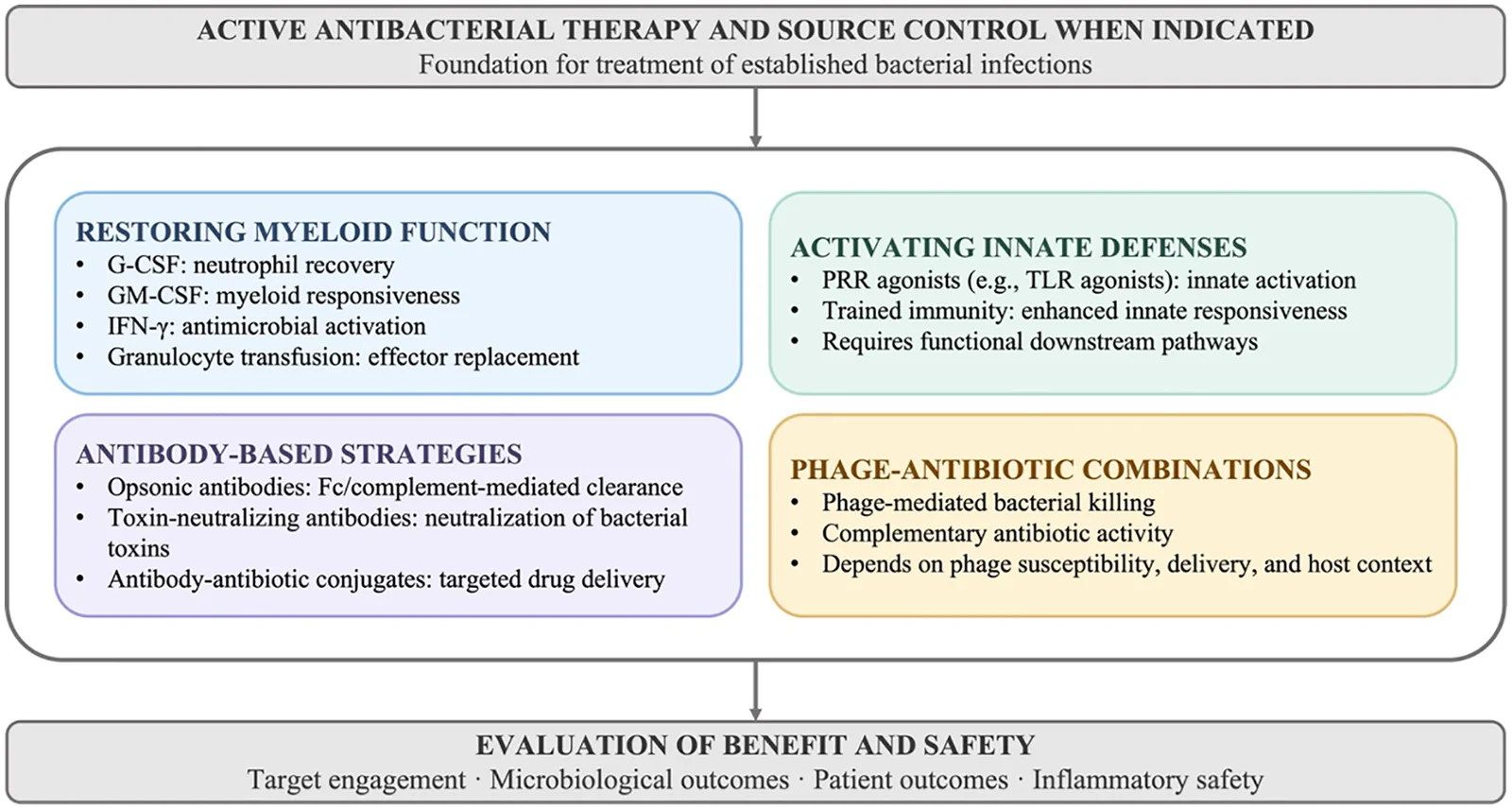
Mechanisms and host requirements of adjunctive strategies.

**Table 3:** Adjunctive strategies and limits of the clinical evidence.

Strategy	Required biological context	Representative evidence	Evidence interpretation	Principal limitation
G-CSF/GM-CSF	Quantitative neutrophil deficiency or a defined monocyte-hyporesponsive phenotype, according to the intervention	G-CSF in high-risk febrile neutropenia; biomarker-guided GM-CSF studies including Meisel and GRID (Garcia-Carbonero et al., 2001; Er et al., 2004; Meisel et al., 2009; Vacheron et al., 2023)	G-CSF shortened neutropenia without a demonstrated mortality benefit; GM-CSF increased mHLA-DR and ex vivo responsiveness.	GRID ended early with insufficient enrollment to resolve its clinical outcome questions.
Interferon-γ	A host defect in which interferon-responsive antimicrobial function is biologically relevant	Randomized trial of interferon-γ prophylaxis in chronic granulomatous disease (International Chronic Granulomatous Disease Cooperative Study, 1991)	Reduced serious infections during CGD prophylaxis.	Evaluated for prevention in CGD, not as treatment of established acute AMR infection.
Granulocyte transfusion	Severe quantitative granulocyte deficiency with an infection in which temporary effector replacement is biologically plausible	RING randomized trial (Price et al., 2015)	No established overall benefit in the randomized trial; remains a specialized intervention	Trial underpowered because of incomplete enrollment; dose-response signal was *post hoc*; logistical and transfusion-related constraints
PRR agonists and trained-immunity approaches	Retained downstream signaling and effector capacity; an anatomical setting in which activation can occur without unacceptable inflammatory injury	MPLA plus amoxicillin; mucosal flagellin; combined TLR2/6–TLR9 stimulation; PAMP augmentation in an immunocompromised wound model; β-glucan training (Munoz et al., 2010; Leiva-Juarez et al., 2016; Casilag et al., 2021; Stothers et al., 2021; Padmakumari et al., 2026)	Preclinical studies demonstrate enhanced antibacterial activity or a durable trained phenotype	Human target engagement and treatment efficacy in acute bacterial infection are unestablished; dose, timing, compartment, inflammatory toxicity, and pathway tolerance are major uncertainties
Toxin-neutralizing antibodies	A clinically relevant toxin that is accessible to antibody neutralization	Bezlotoxumab added to antibacterial treatment for *C. difficile* infection (Wilcox et al., 2017)	Reduced recurrent infection without materially improving initial cure; benefit is endpoint-specific	Evidence concerns toxin B neutralization and recurrence prevention rather than direct antibacterial activity.
Other pathogen-specific antibodies	Accessible antigen; adequate site exposure; residual Fc-, complement-, or phagocyte-mediated function when required by the mechanism	Suvratoxumab and gremubamab prevention programs; AR-301 treatment study; experimental panobacumab (Secher et al., 2013; Francois et al., 2018; Francois et al., 2021; Chastre et al., 2022)	Clinical results are mixed and prevention and treatment evidence are not interchangeable; benefit is intervention-, indication-, and endpoint-specific	Antigen heterogeneity, target accessibility, site exposure, timing, population enrichment, and host-effector dependence
Antibody–antibiotic conjugates	Pathogen recognition, cellular uptake, and intracellular payload release	Preclinical intracellular *S. aureus* clearance; healthy-volunteer phase 1; published phase 1b in complicated SAB (Lehar et al., 2015; Peck et al., 2019; Miller et al., 2026)	Phase 1b identified infusion-related reactions and lower exposure than in healthy volunteers; further development for SAB is not planned.	Significant immune suppression was excluded (ClinicalTrials.gov, 2020); the small trial primarily assessed safety and PK
Phage–antibiotic combinations	Isolate susceptibility, adequate site delivery, and relevant host context; complementary host effectors may contribute in some systems	Experimental phage–antibiotic synergy and host dependence; case evidence of treatment-associated phage neutralization (Oechslin et al., 2017; Abd El-Aziz et al., 2019; Dedrick et al., 2021; Weissfuss et al., 2025)	Experimental drug–phage interactions and immune-dependent activity; clinical evidence remains limited.	Strain specificity, evolving phage resistance, host-mediated neutralization, biodistribution, and poor access or amplification in abscesses, biofilms, or other protected compartments

CGD, chronic granulomatous disease; G-CSF, granulocyte colony-stimulating factor; GM-CSF, granulocyte-macrophage colony-stimulating factor; mHLA-DR, monocyte human leukocyte antigen-DR; MPLA, monophosphoryl lipid A; PAMP, pathogen-associated molecular pattern; PRR, pattern-recognition receptor; SAB, *Staphylococcus aureus* bacteremia; TLR, Toll-like receptor.

Adjunctive strategies target different aspects of host defense and bacterial survival. For established bacterial infections, active antibacterial therapy and source control when indicated remain the clinical foundation. Myeloid-directed approaches aim to restore neutrophil availability or cellular antibacterial function. Innate immune agonists and trained-immunity approaches seek to enhance antibacterial responses through functional host pathways. Antibody-based strategies promote opsonization, neutralize bacterial toxins, or deliver antibiotic payloads to targeted bacterial populations. Phage–antibiotic combinations provide pathogen-directed antibacterial activity influenced by bacterial susceptibility, infection-site delivery, and host immune function. Evaluation of these strategies should consider target engagement, microbiological outcomes, patient outcomes, and inflammatory safety. Clinical evidence varies by intervention and indication, as detailed in **Table 3**.

### 7.1 Restoring myeloid cell number or function

Colony-stimulating factors, interferons, and cell transfusions may increase myeloid-cell numbers or function, but their effects depend on the specific immune defect. In high-risk febrile neutropenia, adjunctive G-CSF shortened severe neutropenia, antibiotic treatment, and hospitalization without reducing mortality, whereas a smaller meropenem-based trial did not significantly improve fever resolution, hospitalization, or mortality (Garcia-Carbonero et al., 2001; Er et al., 2004).

In patients selected for sustained low mHLA-DR, GM-CSF increased mHLA-DR and restored ex vivo TLR2/4-induced cytokine responsiveness in the Meisel trial; mechanical ventilation duration was also shorter (Meisel et al., 2009). In GRID, GM-CSF increased mHLA-DR but did not demonstrate a reduction in ICU-acquired infection or 28-day mortality. GRID ended early because of insufficient recruitment, limiting its ability to resolve clinical outcome questions (Vacheron et al., 2023).

In chronic granulomatous disease, prophylactic interferon-γ reduced serious infections despite no significant improvement in measured superoxide production (International Chronic Granulomatous Disease Cooperative Study Group, 1991).

Granulocyte transfusion aims to replace deficient effector cells. The randomized RING trial did not improve its composite endpoint of survival and microbial response, although incomplete enrollment reduced power; an exploratory dose-response signal arose from a *post hoc* analysis (Price et al., 2015). Granulocyte transfusion consequently remains a specialized intervention.

### 7.2 Innate agonists, epithelial defense, and trained immunity

Pattern-recognition receptor agonists aim to activate innate antibacterial defenses rapidly. In murine pneumococcal pneumonia, monophosphoryl lipid A (MPLA) plus amoxicillin reduced bacterial burden and improved survival beyond either intervention alone (Casilag et al., 2021). Mucosal flagellin enhanced protection through TLR5-dependent neutrophil responses (Munoz et al., 2010), whereas combined TLR2/6–TLR9 stimulation strengthened epithelial defense in leukemia- and chemotherapy-associated pneumonia models (Leiva-Juarez et al., 2016). In the tobramycin–*Pseudomonas aeruginosa* wound model, prophylactic LPS or fMLP improved subsequent bacterial control in immunodeficient mice (Padmakumari et al., 2026).

These strategies can target host-response pathways independently of a specific resistance determinant, but the same signaling that promotes antibacterial activity can also cause inflammatory injury. Dose, timing, burden, and anatomical compartment are therefore critical, and repeated LPS/TLR4 stimulation can produce pathway-specific hyporesponsiveness or tolerance (Peng et al., 2012). Development should consequently favor controllable exposure, compartment-appropriate delivery where feasible, active antibacterial background therapy, and prespecified measures of antibacterial target engagement and inflammatory toxicity.

Trained immunity describes lasting changes in innate immune responses after an initial stimulus. β-Glucan training increased innate-cell recruitment and *P. aeruginosa* clearance in mice, with persistent protection and transferable macrophage activity (Stothers et al., 2021). These preclinical findings motivate studies of trained immunity in defined AMR populations, including the durability of antibacterial activity and the risk of inflammation.

### 7.3 Antibodies and targeted antibacterial delivery

Monoclonal antibodies can neutralize toxins or virulence determinants, promote Fc- or complement-dependent clearance, or deliver antimicrobial payloads. Their specificity may limit nonspecific immune stimulation but makes efficacy dependent on pathogen and antigen identification, target accessibility, infection-site exposure, and, for opsonic antibodies, sufficient downstream host effector function.

Bezlotoxumab added to antibiotics reduced recurrent *Clostridioides difficile* infection through toxin B neutralization, while initial cure was essentially unchanged (Wilcox et al., 2017).

In prevention trials, suvratoxumab produced a numerical but nonsignificant reduction in *Staphylococcus aureus* pneumonia among colonized mechanically ventilated patients (Francois et al., 2021). Gremubamab did not reduce *Pseudomonas aeruginosa* pneumonia, although many recipients exceeded a serum exposure threshold derived from animal models (Chastre et al., 2022). In established severe *S. aureus* pneumonia, AR-301 showed initial safety and pharmacokinetic feasibility without establishing definitive clinical efficacy (Francois et al., 2018). Population selection, target accessibility, site exposure, and treatment timing may contribute to these different results.

Panobacumab improved bacterial clearance in experimental O11 *Pseudomonas aeruginosa* pneumonia. It had additive activity with meropenem against a susceptible strain and retained activity against a meropenem-resistant strain, in which the combination did not show an additional benefit (Secher et al., 2013). Its activity is limited to the O11 serotype.

Antibody–antibiotic conjugates provide a strategy for intracellular delivery (Lehar et al., 2015). DSTA4637S underwent healthy-volunteer safety/PK evaluation (Peck et al., 2019), followed by a published phase 1b study in complicated *S. aureus* bacteremia. Four of 19 DSTA4637S recipients discontinued treatment because of infusion-related reactions, and systemic exposure was 30–60% lower than in healthy volunteers. Further development for bacteremia is not planned (Miller et al., 2026). These findings identify safety and exposure barriers to clinical translation.

### 7.4 Phage combinations and host dependence

Bacteriophages are pathogen-directed antibacterial agents rather than immune adjuvants, but their *in vivo* activity can depend on host effectors. In *P. aeruginosa* endocarditis, phage plus ciprofloxacin produced synergistic *in vivo* killing and suppressed regrowth of phage-resistant subpopulations in fibrin-clot experiments (Oechslin et al., 2017). In experimental multidrug-resistant (MDR) *P. aeruginosa* lung infection, phage therapy improved bacterial clearance and survival, while complementary *in vitro* experiments showed enhanced complement-mediated lysis of phage-exposed bacteria (Abd El-Aziz et al., 2019). More recently, neutrophils substantially enhanced pulmonary phage-mediated bacterial control, particularly at high burden, whereas alveolar-macrophage depletion had little effect (Weissfuss et al., 2025). These studies identify complement and neutrophils as modifiers of phage-mediated bacterial control in the tested models.

Clinical development should therefore characterize host factors likely to modify phage activity, including neutrophil status, complement-modifying therapy, bacterial burden, and treatment timing, and should not assume that broth phage susceptibility predicts *in vivo* efficacy. During repeated or prolonged phage treatment, adaptive immune responses may also reduce phage activity. In a six-month intravenous phage treatment for refractory *Mycobacterium abscessus* infection, an initial microbiological response was followed by IgM- and IgG-mediated phage neutralization coinciding with loss of benefit (Dedrick et al., 2021). Anti-phage antibody responses vary with the phage preparation, administration route, treatment duration, prior exposure, and host immune state; their relationship to clinical outcomes remains inconsistent (Washizaki et al., 2024).

Phage–antibiotic combinations face additional barriers to translation. Phage distribution depends on administration route, clearance, immune sequestration, bacterial density, and replication at the infection site (Kang et al., 2024; Siopi et al., 2024). Delivery and amplification may be uneven within abscesses, necrotic tissue, and biofilms, where antibiotics and immune cells also have restricted access. Phage susceptibility varies among isolates of the same bacterial species and can change over time, supporting testing of contemporaneous isolates (Martin et al., 2021; Kolenda et al., 2024). Clinical studies should therefore assess contemporaneous isolate susceptibility, the specific drug–phage combination, site delivery, and longitudinal emergence of phage resistance or host-mediated neutralization.

### 7.5 Matching adjuncts to specific immune defects

For each proposed adjunct, researchers should identify its target, how it works, and which host functions it needs to remain effective. Cell-number restoration, monocyte activation, opsonization, intracellular delivery, toxin neutralization, and disruption of protected architecture are distinct objectives. In transplant recipients, adjunct selection must also account for graft protection and the consequences of altering immunosuppression.

## 8 Priorities for translation in immunocompromised populations

The proposed framework integrates antibiotic exposure, bacterial phenotype, host immune function, and infection-site conditions to guide mechanistic investigation and identify priorities for clinical research.

### 8.1 Reassessment of persistent infection

Persistent infection offers a clinical setting in which antibiotic exposure, bacterial phenotype, infection anatomy, and immune recovery can be studied together (**Figure 1**). Serial microbiology should establish ongoing infection and phenotype changes; exposure assessment should document active treatment and drug delivery; source assessment should describe protected foci; and host assessment should follow the relevant immune deficit over time.

In persistent CRE bacteremia during chemotherapy-associated neutropenia, serial cultures, current susceptibility, active antibiotic delivery, source control, and infection-site exposure can be aligned with the neutrophil trajectory to examine whether bacterial persistence changes with immune recovery.

For a liver transplant recipient with CRKP and suspected intra-abdominal infection, culture interpretation, drainage, drug exposure, organ function, and immunosuppressive treatment provide a different set of study variables. Persistent anatomical sources and graft-related constraints on immunosuppression can be evaluated alongside bacterial clearance.

### 8.2 Measure host and pathogen trajectories together

Serial measurements matched to the suspected immune deficit offer a more informative picture of host function than a single biomarker result. For neutrophil-mediated clearance, cell-count trajectories may be complemented in development studies by assays of cellular function or tissue recruitment. For monocyte-directed therapy, standardized mHLA-DR reported as antibodies bound per cell has shown interlaboratory reproducibility and has been validated for defined clinical-trial enrollment use (Demaret et al., 2013; Quadrini et al., 2021); complementary stimulation assays can assess cellular responsiveness. For complement-dependent strategies, pathway activity, complement-modifying therapy, and, where relevant, pathogen susceptibility to serum-mediated killing may be informative. Local cytokine or injury markers may be more relevant than systemic leukocyte counts for epithelial or mucosal interventions.

Prospective studies should record the type and timing of immune impairment rather than simply classify patients as immunocompromised or not. Hematologic malignancy, hematopoietic cell transplantation, solid-organ transplantation, defined phagocyte defects, and complement-modifying treatment provide distinct study populations. Routine measurements can include blood counts, medication exposures, microbiology, source-control timing, and organ function; experimental assays should be selected for a stated biological question. Resistant and susceptible infections should be distinguished, and pathogen genotype or phenotype assessed where it may modify the proposed interaction.

Prognostic biomarkers and markers that predict response to an adjunct should be evaluated separately. Predictive studies should test whether the marker identifies treatment-response differences beyond established clinical factors. Longitudinal immune-by-exposure or immune-by-intervention analyses require adequate sample size, prespecified timing, and consideration of time-varying confounding.

### 8.3 Use models that preserve the relevant immune defect

Experimental models should standardize key determinants of antibacterial response, including the pathogen, inoculum, infection site, treatment timing, and antibacterial exposure (Arrazuria et al., 2022). Candidate antibacterial–adjunct combinations should therefore be evaluated in paired immune-state designs that hold the pathogen, inoculum range, site, treatment timing, and antibacterial exposure as comparable as feasible, with consistent exposure measurement. When feasible, models with several levels of immune impairment are preferable to a simple impaired-versus-intact comparison because they can reveal effects that change with immune function, as illustrated by PK/PD studies comparing neutropenic, intermediate, and immunocompetent states (Saporta et al., 2026). Host-state manipulations or phenotypes should also be mechanistically defined rather than treated as interchangeable; neutrophil depletion, marrow suppression, monocyte hyporesponsiveness, complement blockade, and chemotactic dysfunction represent distinct host deficits.

Paired studies should include clinically relevant resistant isolates as well as well-characterized reference strains. Where feasible, factorial designs should compare antibiotic alone, adjunct alone, their combination, and untreated controls across defined host states, with measured site exposure and longitudinal burden. Quantitative interaction analysis is needed before a combination is called synergistic. Complementary testing in organotypic, serum, whole-blood, and intracellular systems could establish whether candidate mechanisms remain active under relevant biological conditions. Studies of resistant pathogens should also include longitudinal assessment of bacterial burden, antibiotic susceptibility, and resistance-associated mutations across defined immune states. These measurements can help distinguish impaired bacterial clearance from the selection of progressively less susceptible populations during treatment, as illustrated by experimental evolution in a mouse pneumonia model (Huo et al., 2022).

Combined models can examine how antibiotic concentration, bacterial burden, immune activity, and tissue injury change together. Drug- and host-attributable effects may be difficult to identify from observational data alone, so model development needs informative perturbations and external validation. Site-specific and intracellular exposures should be assessed where the proposed mechanism requires them (Brunner et al., 2005; Mouton et al., 2008; Saporta et al., 2026).

### 8.4 Test added benefit and inflammatory safety

Trials should define the target infection or prevention setting and the biological context the adjunct is intended to modify, including a functional host deficit where relevant. Prevention and treatment studies should be distinguished explicitly. For treatment of established infection, the adjunct should be evaluated against an appropriate active antibacterial background. Early studies should establish delivery, safety, and target engagement. Subsequent endpoints should match the mechanism and may include incident infection, sustained culture clearance, recurrence, secondary infection, organ support, or survival.

The need for functioning host pathways also affects which patients might benefit: an opsonic antibody may require residual downstream effectors, while the activity of some phage combinations may be modified by complementary host pathways; an immune agonist requires a responsive pathway and a tolerable inflammatory window. Immunocompromised populations should therefore be included or excluded for a stated mechanistic reason, with their representation reported explicitly.

Inflammatory toxicity must be studied with the intended antibiotic, burden, timing, and compartment. Antibiotic-mediated injury can alter microbial-product exposure, and increased cytokine signaling can accompany either improved clearance or tissue damage. Both local activity and systemic spillover matter for compartment-directed interventions. **Table 4** summarizes the study priorities, and **Supplementary Figure S3** shows the proposed development pathway.

**Table 4:** Research priorities for defined immunocompromised populations.

Priority	Design requirement	Measurements	Criterion for progression
Define host and infection	Specify disease, treatment, timing, immune trajectory, infection compartment, and pathogen/resistance phenotype	Microbiology, medications, immune deficit, and infection compartment	Reproducible phenotype and infection classification
Paired immune-state models	Hold pathogen, site, burden, timing, and antibacterial exposure comparable	Longitudinal bacterial counts, recruitment/function, and injury	Interaction persists across relevant conditions and isolates
Preserve biological context	Use relevant blood, serum, cells, or structured tissue models	Functional complement, intracellular burden, microbial-product activity	Mechanism persists in relevant biological matrices
Confirm site exposure	Measure active antibacterial exposure and adjunct exposure or target engagement in the relevant compartment where feasible	Plasma, tissue, and intracellular exposure or target engagement	Biologically active exposure with acceptable toxicity
Qualify functional biomarkers	Standardize sampling; test intended use prospectively	Host, drug, burden, and injury trajectories	Adds information beyond established factors and, when intended for treatment selection, demonstrates treatment-response heterogeneity
Validate quantitative models	Use informative perturbations and external datasets	Identifiability and predictive performance	Incremental value over simpler models
Evaluate adjunctive benefit	Define the prevention or treatment setting and the biological context the adjunct is intended to modify; for established infection, use an appropriate active antibacterial background	Safety, target engagement, incident infection or sustained clearance as appropriate, and patient outcomes	Acceptable safety and a justified infection-related signal
Assess inflammatory safety	Test the intended antibiotic, burden, timing, and route	Local/systemic inflammation, vascular and organ injury	A measurable window between control and harm

## 9 Discussion

This Review links antibiotic-induced bacterial injury with the innate immune functions required for bacterial clearance. By considering antibacterial exposure, phagocyte and complement activity, bacterial burden, and protected infection compartments together, it identifies mechanisms that may contribute to persistent infection despite apparently active treatment. These interactions generate specific research questions, including how impaired host clearance modifies antibacterial exposure–response relationships, how infection-site conditions influence antibiotic–immune interactions, and which functional deficits may be suitable targets for adjunctive interventions.

The available evidence has important gaps. Clinical AMR cohorts establish resistant infection and adverse outcomes in hematologic malignancy and transplantation, but generally lack integrated measurements of host immune function, antibiotic exposure, and longitudinal bacterial burden needed to characterize drug–host interactions (Satlin et al., 2016; Dang et al., 2026). Experimental studies provide stronger mechanistic separation of drug and host effects, but generally examine few strains, short time courses, and simplified immune perturbations (Thorsted et al., 2023; Saporta et al., 2026). Few studies have tested whether correcting a measured immune deficit improves outcomes of resistant bacterial infection in a defined immunocompromised population.

This structured narrative Review did not attempt exhaustive study identification or conduct formal risk-of-bias or certainty-of-evidence assessments. Selective literature coverage and heterogeneity across studies may therefore influence the synthesis. Human observational data also cannot readily separate immune dysfunction from changes in disease severity, antibiotic exposure, and source-control opportunities. The proposed framework has not undergone clinical validation; its premature application to individual antibiotic dosing or adjunctive treatment selection could lead to inappropriate treatment decisions.

Future studies should test whether measuring a specific immune function improves interpretation of antibiotic response at a defined infection site and whether an appropriately targeted intervention improves sustained bacterial clearance or patient outcomes.

## 10 Conclusion

Innate immune deficits can modify bacterial containment and clearance during antibiotic treatment, but their effects depend on the pathogen, bacterial burden, and infection compartment. For immunocompromised patients with resistant bacterial infection, priorities remain effective antibiotic treatment, adequate drug exposure, assessment and control of the infection source when indicated, and monitoring of immune recovery. Prospective studies should determine when a defined immune deficit modifies antibiotic response and whether targeted adjuncts improve sustained bacterial clearance or patient outcomes.
