# Supplementary material for: Innate immunity and antibiotic activity in resistant bacterial infections in immunocompromised hosts
Source: Front Cell Infect Microbiol. 2026 Oct 29;16:2002377. doi: 10.3389/fcimb.2026.2002377 (PMC13756140; doi:10.3389/fcimb.2026.2002377)
Supplement: Supplementary file 1 [file DataSheet1.docx]

***Supporting Information***

Supplementary Figure S1. Resistance, tolerance, and persistence during antibiotic exposure.
Resistance permits growth at higher antibiotic concentrations and is reflected by an increased minimum inhibitory concentration (MIC). Tolerance denotes slower killing of the bacterial population without an increased MIC; persistence denotes prolonged survival of a subpopulation during exposure to bactericidal antibiotics without an increased MIC (Balaban et al., 2019). These phenotypes can coexist. Innate effectors may help clear antibiotic-injured or surviving bacteria through recognition, phagocytosis, and intracellular killing, with their contribution depending on bacterial phenotype, burden, and tissue access.

**Supplementary Figure S2. Protected compartments and residual bacterial survival.**
Intracellular reservoirs and extracellular biofilm or abscess communities represent distinct, potentially overlapping forms of bacterial protection during antibiotic exposure. Intracellular survival depends on host-cell and subcellular location, active drug access, and bacterial physiological state. Biofilm and abscess communities combine spatial organization, heterogeneous physiology, and restricted access by antibiotics and immune effectors. Either compartment may contain resistant bacteria, tolerant populations, or persister subpopulations. Biofilm architecture describes spatial organization, while tolerance and persistence describe killing phenotypes.

**Supplementary Figure S3. Proposed translational pathway for host–antibiotic interventions.**
Paired or graded host-state models support mechanism and biomarker identification, joint analysis of antibiotic exposure, bacterial burden and immune function, clinical host phenotyping, and trials of mechanistically justified combinations. Progression requires evidence of site delivery, target engagement, antibacterial activity, and inflammatory safety. Unmet criteria prompt reassessment of the mechanism, delivery, timing, population, or model assumptions.

***References***

Balaban, N.Q., Helaine, S., Lewis, K., Ackermann, M., Aldridge, B., Andersson, D.I., et al. (2019). Definitions and guidelines for research on antibiotic persistence. *Nat Rev Microbiol* 17(7)**,** 441-448. doi: 10.1038/s41579-019-0196-3.
